# Genome-Wide Identification of *AhMDHs* and Analysis of Gene Expression under Manganese Toxicity Stress in *Arachis hypogaea*

**DOI:** 10.3390/genes14122109

**Published:** 2023-11-21

**Authors:** Ying Liu, Min Zhao, Jianning Shi, Shaoxia Yang, Yingbin Xue

**Affiliations:** 1 Department of Biotechnology, College of Coastal Agricultural Sciences, Guangdong Ocean University, Zhanjiang 524088, China; liuying85168@gdou.edu.cn (Y.L.); 15295513968@163.com (J.S.); 2 Department of Agronomy, College of Coastal Agricultural Sciences, Guangdong Ocean University, Zhanjiang 524088, China

**Keywords:** *Arachis hypogaea*, MDH, genome-wide identification, expression analysis, manganese toxicity stress

## Abstract

Malate dehydrogenase (MDH) is one kind of oxidation–reduction enzyme that catalyzes the reversible conversion of oxaloacetic acid to malic acid. It has vital functions in plant development, photosynthesis, abiotic stress responses, and so on. However, there are no reports on the genome-wide identification and gene expression of the MDH gene family in *Arachis hypogaea*. In this study, the MDH gene family of *A. hypogaea* was comprehensively analyzed for the first time, and 15 AhMDH sequences were identified. According to the phylogenetic tree analysis, *AhMDHs* are mainly separated into three subfamilies with similar gene structures. Based on previously reported transcriptome sequencing results, the *AhMDH* expression quantity of roots and leaves exposed to manganese (Mn) toxicity were explored in *A. hypogaea*. Results revealed that many *AhMDHs* were upregulated when exposed to Mn toxicity, suggesting that those *AhMDHs* might play an important regulatory role in *A. hypogaea*’s response to Mn toxicity stress. This study lays foundations for the functional study of *AhMDHs* and further reveals the mechanism of the *A. hypogaea* signaling pathway responding to high Mn stress.

## 1. Introduction

MDH (malate dehydrogenase) is one of the oxidation–reduction enzymes widely present in plants, animals, and microorganisms, and it has highly conserved properties [1]. MDH relies on the NADH/NADPH cofactor to participate in the biochemical reaction of the reversible conversion of oxaloacetic acid to malic acid [2,3]. According to the different cofactors on which MDH depends, MDH contains two categories: NAD-MDH and NADP-MDH, among which NAD-MDH is mainly present in the cytoplasm, mitochondria, glyoxysome, peroxidation object, and chloroplast, while NADP-MDH only exists in the chloroplast [4,5]. MDH can participate in the regulation of oxaloacetate changing to malate and NAD^+^ to NADH, providing reducing power for the nitric acid reduction process [6,7]. NADH produced through biological oxidation in the cytosol cannot pass through the inner mitochondrial membrane [8,9]. However, NADH can deliver hydrogen to oxaloacetic acid under the action of MDH to produce malic acid, which can be transported to the mitochondrial matrix through the malate–aspartate shuttle system [8,9]. Furthermore, the malic acid in the mitochondrial matrix is reoxidized to oxaloacetic acid and NADH and then oxidized through the respiratory chain [8,9]. NADP-MDH is a light-activated enzyme participating in the regulation of C_4_ photosynthesis, and NADP-MDH is also light-activated in C_3_ plants; however, NADP-MDH is relatively inactive in the dark [10].

Plant MDH is involved in a variety of metabolic pathways, such as the tricarboxylic acid cycle, the glyoxylic acid cycle, amino acid metabolism, redox equilibrium, and gluconeogenesis [11]. MDH is closely associated with different organelles through the malic acid shuttle system [12,13]. The malic acid in cells is indirectly transferred to different organelles, thus maintaining the physiological and biochemical metabolism balance in plants and playing an important part in the exchange of substances between the organelles and cytosol [12,13]. Furthermore, many scientific studies reports have shown that plant MDHs play vital parts in seed growth, photosynthesis and coping with abiotic stress and biological stress [14].

With the sequencing of whole genomes and the development of bioinformatics, many types of MDHs have been identified in different plants: 12 MDHs have been found in rice (*Oryza sativa*) [15], 16 MDHs have been discovered in soybean (*Glycine max*) [16], 12 MDHs have been found in Chinese fir (*Cunninghamia lanceolata*) [13], 13 MDHs have been identified in cotton (*Gossypium hirsutum*) [17], 7 MDHs have been detected in Stylosanthes (*Stylosanthes guianensis*) [18], 20 MDHs have been discovered in apple (*Malus pumila*) [19], and 16 MDH genes have been found in poplar (*Populus trichocarpa*) [20].

Peanut (*Arachis hypogaea*) is one of the main oil crops in tropical and subtropical areas [21]. It provides edible oil and edible protein for people worldwide and accounts for 30% of China’s total oilseed production [22]. Peanut is rich in protein, fat, carbohydrates, and vitamins, with a fat content of 44–45% and a protein content of 24–36%, making it one of the most widely consumed nuts in China [23,24]. The nutritional value of peanut is high, and it is easily absorbed and utilized by the human body, making it comparable to animal foods such as eggs, milk, and lean meat [25].

Seeds with vigorous vitality and moderate dormancy guarantee seedling size and seedling strength after planting and directly affect peanut yield [26]. During the process of water absorption and the expansion of seeds, mitochondrial respiration is rapidly enhanced, malate dehydrogenase activity increases, and stored substances in seeds are mobilized to provide energy for seed germination [27]. The decreased activity of malate dehydrogenase and cytochrome C oxidase may lead to an insufficient mitochondrial energy supply and the accumulation of reactive oxygen species (ROS), leading to decreased or even a loss of seed vitality [28,29]. Some research reports have found that AhMDH1 encodes mitochondrial malate dehydrogenase, which is a hydrophobic protein, and the gene expression is strongly linked with the water absorption process during seed germination, indicating that MDH is necessary for the mobilization of storage substances during seed germination [30].

Manganese (Mn) is an essential element in almost all living organisms and can play different functional roles in plants, either as an enzyme cofactor or as a catalytically active metal in biological clusters [31]. Mn plays vital parts in photosynthesis, respiration, protein biosynthesis, and hormone activation in plants [32]. When the available Mn content in the soil exceeds the normal Mn concentration needed by a plant, the plant is likely to suffer from Mn toxicity. Plants, as a result, display obvious signs of poisoning on their leaves, such as dark patches, crinkled leaves, and chlorosis; Mn poisoning also affects roots, resulting in reduced lateral root count and dry weight, in addition to the signs on the leaves [33]. Excess Mn prevents the absorption and transfer of other essential elements, such as calcium, magnesium, iron, and phosphorus, possibly because of the similar ionic radii or binding strength of ligands [34]. At the molecular level, excess Mn can prevent the absorption and transfer of other essential elements, such as calcium, magnesium, iron, and phosphorus [35], and inhibit the synthesis of chlorophyll [34], resulting in a decrease in the rate of photosynthesis [36]. The transcriptome sequencing results of peanut roots and leaves treated with 10 and 300 μM of manganese sulfate show that the peanut MDH gene family was strongly induced by manganese toxicity stress, and the expression levels changed significantly [37]; these results need to be further verified through fluorescent quantitative PCR. However, the role of the MDH gene family in Mn toxicity stress in peanut has not been reported.

The MDH gene in the peanut genome has not yet been fully elucidated, and its function needs to be verified. In this study, *AhMDHs* in the genome of peanut were identified using multi-analysis. The analysis of the basic information of *AhMDHs* is helpful to elucidate the molecular regulation mechanisms of peanut development and response to Mn toxicity stress, providing a theoretical grounding for the molecular breeding of peanut.

## 2. Materials and Methods

### 2.1. Analysis of AhMDH Structures

The peanut genome was sourced from the website of National Center of Biotechnology Information (NCBI) (https://www.ncbi.nlm.nih.gov/) (accessed on 1 October 2023), and the transcriptome data (NCBI accession number: PRJNA901194) [37] were used for MDH gene identification. Genes including the domain of MDH were called MDH1 to MDH15, and genes that matched the published AhMDH1 sequence [30] were named AhMDH1. The website of global species databases (GSDs) (http://gsds.gao-lab.org/) (accessed on 2 October 2023) was used based on the annotated information for the peanut genome gff. The structural model of the MDH in peanut was visualized. In this study, TBtools (Toolkit for Biologists integrating various biological data handling tools) [38] was adopted to visualize the structure and intuitively draw the predicted results.

### 2.2. Constructing a Phylogeny Tree of the MDHs

MEGA11 (molecular evolutionary genetics analysis version 11) software [39] was adopted to compare the amino acid sequences of the MDH family of peanut with those of *Arabidopsis thaliana*, *Glycine max*, *Phaseolus vulgaris*, and *Stylosanthes guianensis*. Maximum likelihood estimate (MLE) was used to construct a phylogeny tree of the *MDHs*. Through phylogeny exploration of the peanut *MDHs* and other species, bootstrap analysis was used to build a neighbor-joining (NJ) tree with 1000 repeats. The cutoff value for site coverage was 60%. According to a previous report [40], EOT (Evolview online tools) (https://www.evolgenius.info/evolview/#/treeview) (accessed on 5 October 2023) were used to construct a phylogenetic tree.

### 2.3. Chromosome Localization of the MDH Gene

According to the peanut genome annotation information downloaded from NCBI, the positions of 15 peanut MDH genes on chromosomes were obtained, and TBtools software V1.098 was used to locate the chromosomes [38].

### 2.4. MDH Conservative Motif Analysis

The protein sequence of peanut MDH was presented to the MEME (Multiple Em for Motif Elicitation) (http://meme-suite.org/) (accessed on 6 October 2023) [41] took for the prediction of motifs. The discovery number of the motif displayed was 10; the motif width was set to 6–50. TBtools was used to visualize the MDH motif domain of peanut.

### 2.5. Cis-Acting Element Analysis

Based on the previously reported peanut genome information [20], peanut MDH gene sequences upstream of 2 kb were presented to Plant Care websites (HTML/http://bioinformatics.psb.ugent.be/webtools/plantcare/) (accessed on 8 October 2023) on cis-acting element prediction [42]. The Plant Care analysis results were simplified and visualized using TBTools.

### 2.6. Transcriptome Sequencing Results of AhMDHs in Peanut Roots and Leaves under Manganese Toxicity Stress

The results of the *AhMDHs* in peanut roots and leaves under manganese toxicity stress treatment were derived from RNA sequencing [37]. FPKM (fragments per kilobase of transcript per million mapped reads) values (shown in Table S1) were used to reflect gene expression [43], using the bioinformatics website (http://www.bioinformatics.com.cn/plot_basic_cluster_heatmap_plot_024) (accessed on 9 October 2023) for data visualization.

### 2.7. Peanut Culture Conditions

The peanut variety used was called ZY-62 (Zhanyou 62); it was cultivated by the ZAAS (Zhanjiang Academy of Agricultural Sciences), Guangdong Province, China. The peanut seeds were germinated in quartz sand and cultured for eight days before being treated with manganese. Then, the uniformly growing seedlings of peanut were transferred to plastic incubators with added nutrient solution (volume of 15 L) for water planting [44]. In short, the nutrient solution contained 1500 µM KNO_3_, 400 µM NH_4_NO_3_, 25 µM MgCl_2_, 1200 µM Ca(NO_3_)_2_·4H_2_O, 40 µM Fe-EDTA(Na), 500 µM MgSO_4_·7H_2_O, 0.16 µM (NH_4_)_5_MoO_24_·4H_2_O, 300 µM K_2_SO_4_, 300 µM (NH_4_)_2_SO_4_, 0.5 µM CuSO_4_·5H_2_O, 1.5 µM ZnSO_4_·7H_2_O, 500 µM KH_2_PO_4_, and 2.5 µM NaB_4_O_7_·10H_2_O. All chemicals used were of analytical grade (Kemer, Tianjin, China). Simultaneously, 10 and 300 µM manganese sulfate (MnSO_4_) [37] (Kemer, China) were supplemented to the nutrient solution for manganese treatment. The control group consisted of a nutrient solution containing 10 µM Mn, while the manganese toxicity treatment group contained 300 µM Mn. Every experiment was conducted in 4 biological repetitions. The temperature used to regulate plant development was at 24–29 °C (day)/19–23 °C (night). The period of light was approximately 12 h/d, the light intensity was 2000 lux, and the nutrient solution was renewed every five days. The pH value in the nutrient solution was adjusted to 5.0 with 1 M KOH or H_2_SO_4_ every two days (Kermel, Tianjin, China).

### 2.8. qRT-PCR Analysis of MDH Gene Expression in Peanut

When the peanut seedlings were 20 days old, the roots and leaves were collected, and total RNA was extracted from the peanut leaves and roots using a ribonucleic acid extraction kit (Yisheng, Shanghai, China). After the removal of gDNA (genomic DNA), cDNA was formed by adopting a reverse-transcription kit (Yisheng, China). For conducting quantitative RT-PCR (qRT-PCR) exploration, a fluorescence ration PCR instrument (CFX Connect Optics Module, Bio-Rad, Hercules, CA, USA) was used [37]. In brief, the samples were thinned 30 times for qRT-PCR. The reaction procedure was performed at 95 °C for 30 s, followed by 95 °C for 5 s, 60 °C for 15 s, and 72 °C for 30 s. The internal control gene named *AhUbiquitin* (DQ887087.1) was used to calculate the relativity transcription level based on the transcription ratio of the *AhMDHs* to the *AhUbiquitin*. The primers used for qRT-PCR detection are listed in Table S2.

## 3. Results

### 3.1. Analysis of Basic Information of AhMDHs

All of the 15 studied *AhMDHs* were identified from the whole genome of *A. hypogaea*; 3 of the genes were located on chromosome 09; 2 genes were located on chromosomes 03, 12, and 19; and the other 6 genes were distributed on chromosomes 01, 02, 05, 10, 11, and 13 (Table 1). The CDS length of the *AhMDHs* was approximately 1000 bp, among which *AhMDH2* was the longest, 1359 bp, and its encoded protein was 452 amino acids (Table 1). The relative molecular mass of the AhMDH protein ranged from 35.43 to 47.91 kDa, and the isoelectric points ranged from 6.01 to 9.00. Fifteen *AhMDHs* were predicted; the results showed that AhMDH1 and AhMDH10 were located only in the mitochondrion, and AhMDH3, AhMDH9, and AhMDH14 were located only in the chloroplast. The remaining 10 AhMDHs were located both in mitochondria and chloroplasts (Table 1).

### 3.2. Structural Analysis of AhMDHs

**Figure 1:**
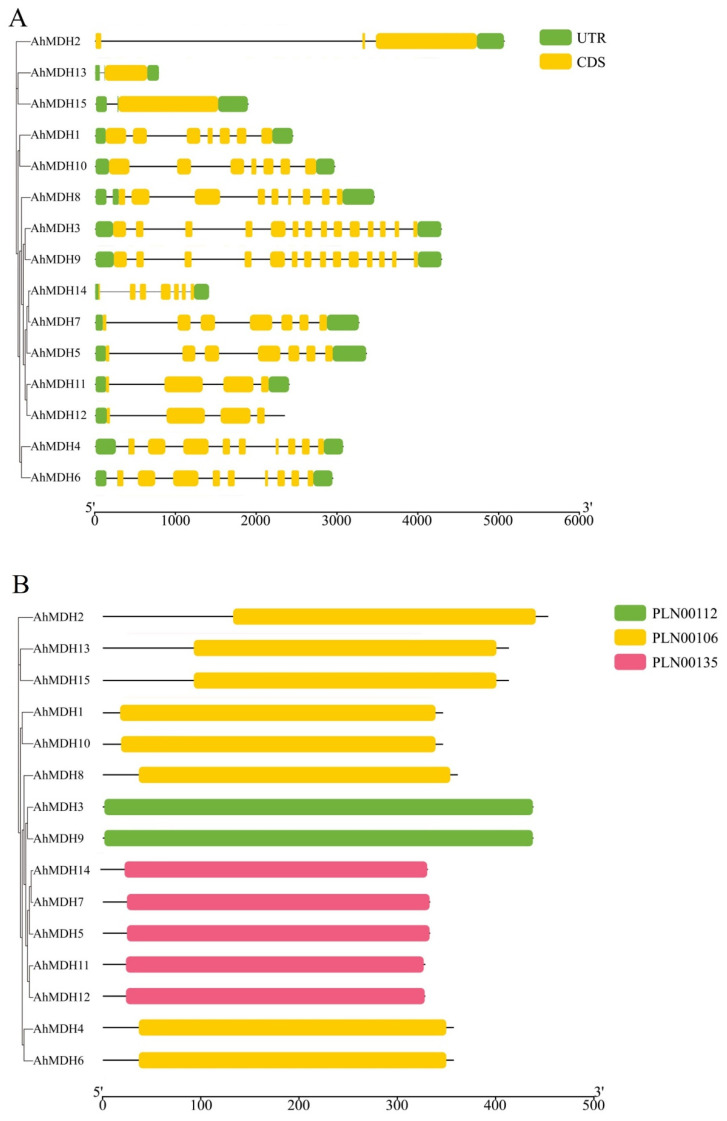
Structure and domain analysis of peanut MDH. (**A**) Genetic structure; (**B**) domain analysis. Conserved protein domain families PLN00112, 00106, and 00135 are classified as models that may span more than one domain.

All 15 MDH genes (*AhMDH1*–*AhMDH15*) were verified in the genome of peanut (Table 1). After analyzing their genetic structure, the research findings displayed that most genes are between 3 kb and 4 kb in length, and most contain more than six exons. The domain prediction revealed that all of the genes contained PLN (plant-specific proteins) domains, and only the number of PLN domains was different (Figure 1).

### 3.3. Evolutionary Tree Analysis of MDHs

**Figure 2:**
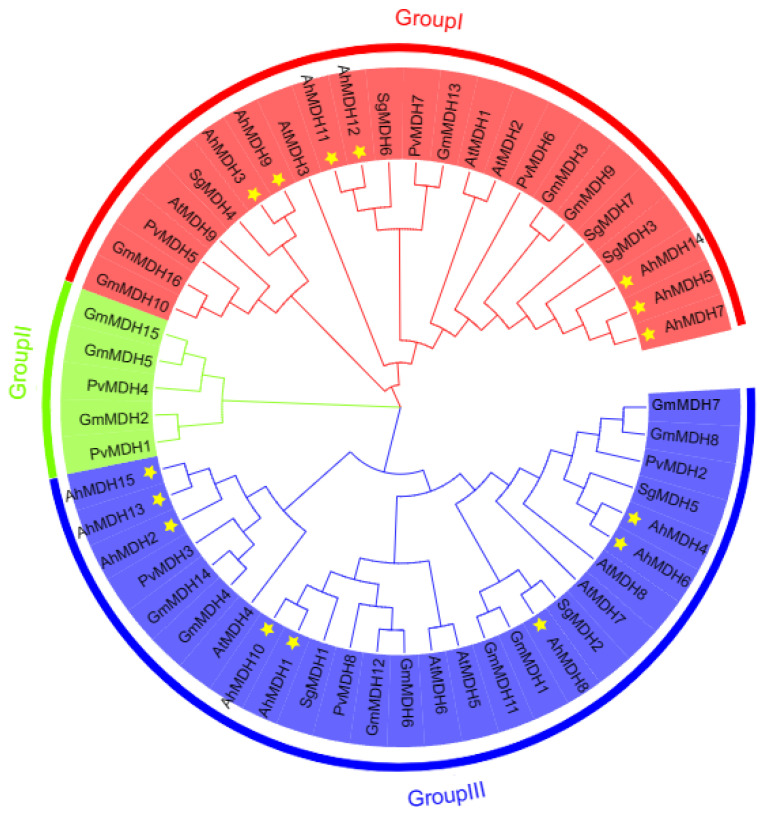
Phylogenetic tree of MDH in *Arachis hypogaea*, *Glycine max*, *Arabidopsis thaliana*, *Phaseolus vulgaris*, and *Stylosanthes guianensis*. *Phaseolus vulgaris*: PvMDH1 (XP_007157340.1), PvMDH2 (XP_007159190.1), PvMDH3 (XP_007155306.1), PvMDH4 (XP_007155646.1), PvMDH5 (XP_007144250.1), PvMDH6 (XP_007145851.1), PvMDH7 (XP_007134537.1), and PvMDH8 (XP_007133189.1); *Arabidopsis thaliana*: AtMDH1 (At1g04410), AtMDH2 (At5g43330), AtMDH3 (At5g56720), AtMDH4 (At3g47520), AtMDH5 (At3g15020), AtMDH6 (At1g53240), AtMDH7 (At2g22780), AtMDH8 (At5g09660), and AtMDH9 (At5g58330); *Stylosanthes guianensis*: SgMDH1 (KJ123727), SgMDH2 (OK188912), SgMDH3 (OK188913), SgMDH4 (OK188914), SgMDH5 (OK188915), SgMDH6 (OK188916), and SgMDH7 (OK188917); *Glycine max*: GmMDH1 (Glyma.01G197700), GmMDH2 (Glyma.01G210400), GmMDH3 (Glyma.02G005500), GmMDH4 (Glyma.05G026300), GmMDH5 (Glyma.05G058100), GmMDH6 (Glyma.06G231500), GmMDH7 (Glyma.07G185400), GmMDH8 (Glyma.08G063800), GmMDH9 (Glyma.10G006500), GmMDH10 (Glyma.10G197700), GmMDH11 (Glyma.11G043900), GmMDH12 (Glyma.12G159300), GmMDH13 (Glyma.13G104800), GmMDH14 (Glyma.17G100600), GmMDH15 (Glyma.17G140600), and GmMDH16 (Glyma.20G192200); *Arachis hypogaea*: AhMDH1 (LOC112790043), AhMDH2 (LOC112718423), AhMDH3 (LOC112776877), AhMDH4 (LOC112791339), AhMDH5 (LOC112711210), AhMDH6 (LOC112728667), AhMDH7 (LOC112776383), AhMDH8 (LOC112800443), AhMDH9 (LOC112711491), AhMDH10 (LOC112737907), AhMDH11 (LOC112735613), AhMDH12 (LOC112726743), AhMDH13 (LOC112710233), AhMDH14 (LOC112711212), and AhMDH15 (LOC112722502). Yellow asterisks indicate the peanut MDH gene.

MEGA11 was used to construct NJ phylogenetic trees of 55 MDH genes from *Arachis hypogaea*, *Glycine max*, *Arabidopsis thaliana*, *Phaseolus vulgaris*, and *Stylosanthes guianensis*. On the basis of the phylogeny tree, peanut MDH was distinguished from three subfamilies (I, II, and III), and the MDH of peanut and *Arabidopsis thaliana* had cross-distribution in subfamilies I and III (Figure 2). There were at most 27 proteins in subfamily III and at least 5 proteins in subfamily II, whereas peanut MDH proteins were only distributed in subfamilies I and III (Figure 2).

### 3.4. Conservative Analysis of Peanut MDH

**Figure 3:**
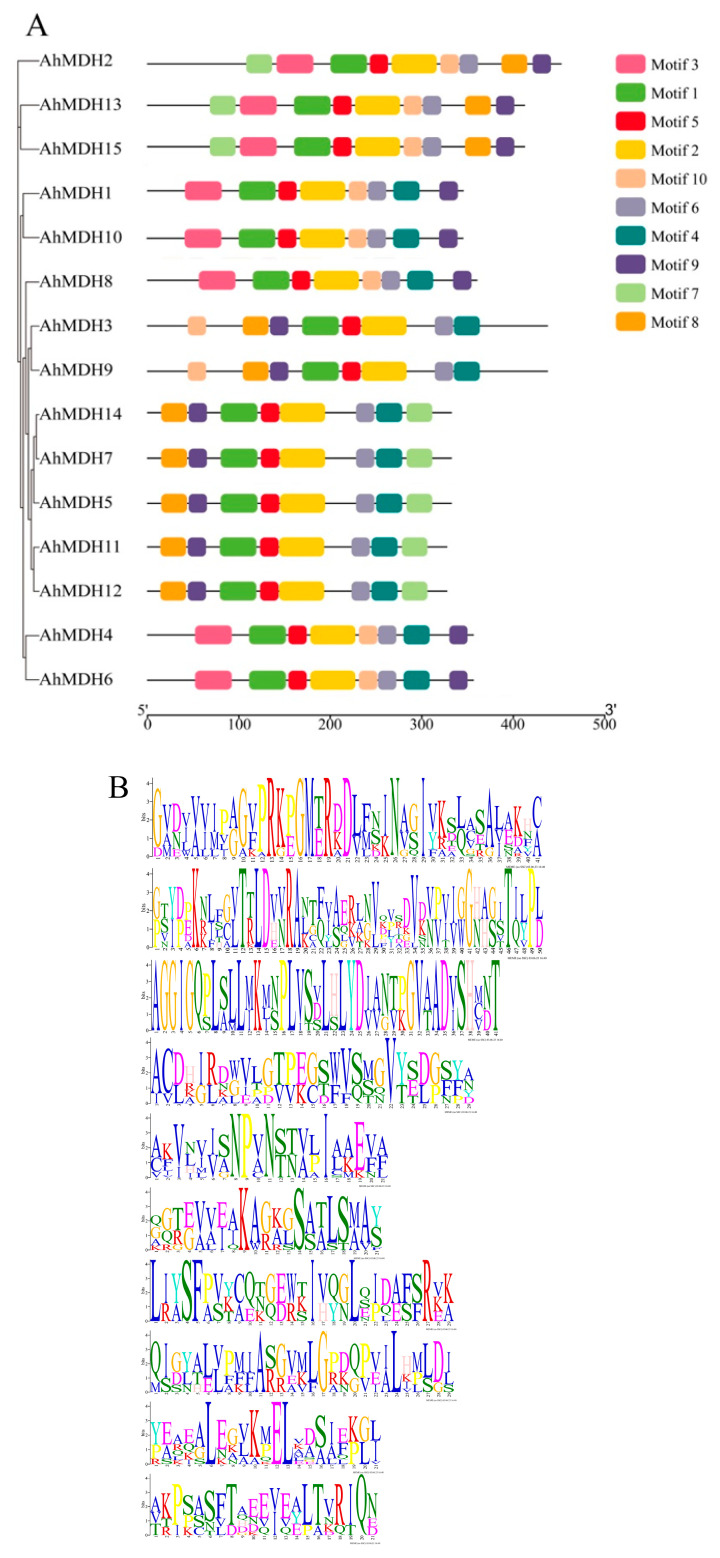
Analysis of MDH gene domain in peanut. (**A**) Domain analysis; different colors represent different motifs, and all genes have motif 1, motif 2, motif 5, and motif 6 domains. (**B**) Motifs analysis; the values are motif 1–motif 10 from top to bottom. Different lengths represent the length of the encoded amino acid.

A conservative motif analysis of peanut MDH sequences was performed using the MEME tool. The amount of MDH motifs in the peanut was basically 8–9 (Figure 3). All genes had motifs 1, 2, 5, and 6, in which motif 1 encoded 41 amino acids, motif 2 encoded 50 amino acids, motif 5 encoded 21 amino acids, and motif 6 encoded 21 amino acids. The amino acid coding sequences created by these four motifs were the key motifs of MDH. Twelve genes contained motif 4, ten genes contained motif 8, and eight genes included motifs 3, 7, 9, and 10. Thus, these domains may play an important role.

### 3.5. Chromosome Location of Peanut MDH

**Figure 4:**
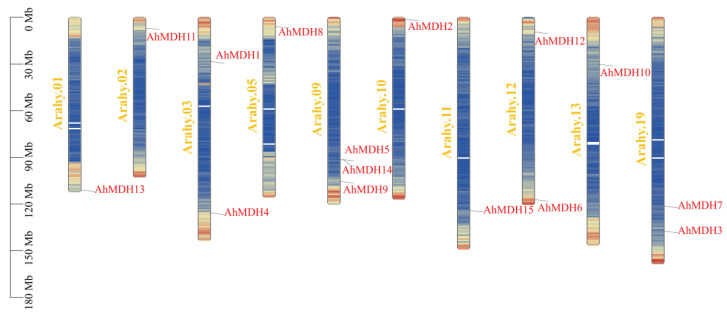
Chromosome location of MDH genes in peanut. Red represents genes and luminous yellow represents chromosome numbers. AhMDH1, AhMDH2, AhMDH3, AhMDH4, AhMDH6, AhMDH8, AhMDH9, AhMDH10, AhMDH11, AhMDH12, and AhMDH13 were located in regions where genes were dense (yellow), while AhMDH5, AhMDH14, AhMDH15, and AhMDH7 were located in regions where genes were dispersed (blue).

Chromosome mapping of the 15 peanut MDH genes was performed (Figure 4). Most of the genes were located in regions where genes were dense (i.e., yellow), while *AhMDH5*, *AhMDH14*, *AhMDH15*, and *AhMDH7* were located in regions where genes were dispersed (blue). According to the results, three genes were situated on chromosome 09, two genes were situated on chromosomes 03, 12, and 19, and only one MDH gene was located on another chromosome. Nine genes were located between 90 and 150 Mb, four genes were located between 0 and 30 Mb (*AhMDH11*, *AhMDH8*, *AhMDH2*, and *AhMDH12*), and the remaining two genes were located between 30 and 60 Mb (*AhMDH1* and *AhMDH10*).

### 3.6. Identification of Cis-Acting Factors of the AhMDHs

**Figure 5:**
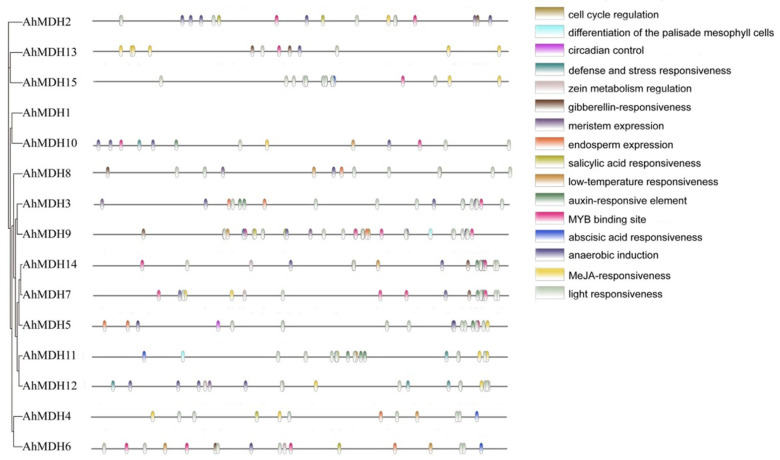
Analysis of cis-acting elements of peanut MDH gene. Homeopathic component distribution.

The length of 2 kb upstream sequences was selected, respectively, from the promoter of 15 *AhMDHs*. The cis-acting elements of the *AhMDHs* promoters were predicted using the PlantCARE online tool. All peanut MDH genes contained 3–11 photoresponsive elements (Figure 5). In addition, the promoter region of the MDH gene in peanut mainly consisted of 46 MeJA (jasmonic acid methyl ester) response elements, 31 anaerobic induction factors, and 30 ABA (abscisic acid) response factors. In addition, there were 20 MYB binding site response elements and a few regulatory factors, such as auxin response factors, salicylic acid response factors, low-temperature response factors, endosperm expression factors, meristem expression factors, erythromycin response factors, zein metabolism regulation elements, stress and defense response factors, circadian rhythm control factors, and cell cycle regulation factors (Figure 5).

### 3.7. Expression Profile of the Peanut MDH Gene

**Figure 6:**
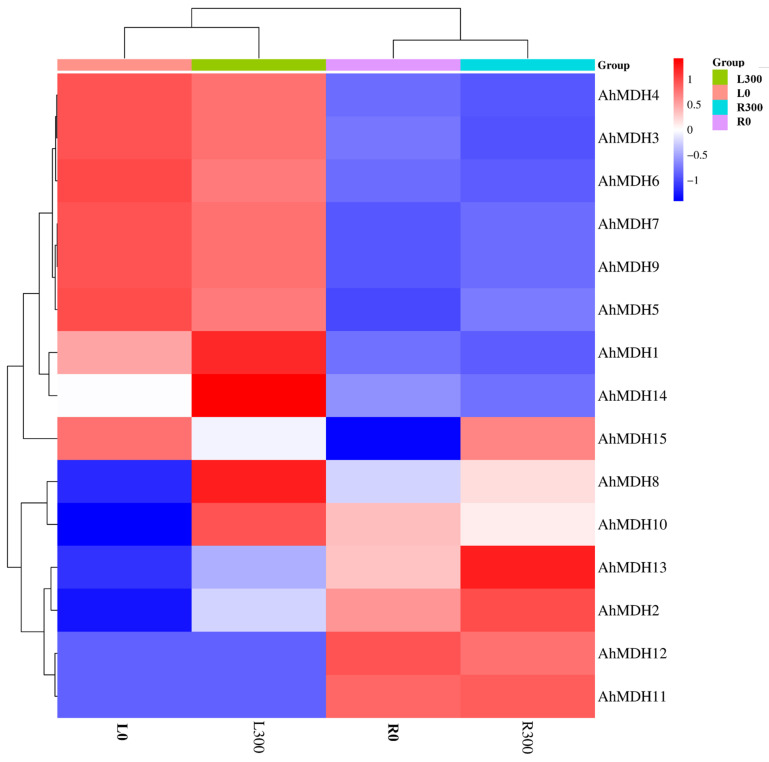
Expression analysis of *AhMDHs* in roots and leaves of peanut treated with manganese detected through transcriptome sequencing. L300: Leaves treated with 300 μM Mn. L0: Leaves treated with 10 μM Mn (control group). R300: Roots treated with 300 μM Mn. R0: Roots treated with 10 μM Mn (control group).

To study the expression profiles of *AhMDHs*, the quantitative analysis of 15 *AhMDHs* expression in roots and leaves treated with Mn were detected through transcriptome sequencing [37]. The experimental results revealed that MDH had a differential expression in both the roots and leaves of peanut (Figure 6). As shown in the Figure 6, *AhMDH4*, *AhMDH3*, *AhMDH6*, *AhMDH7*, *AhMDH9*, *AhMDH5*, *AhMDH1*, and *AhMDH14* were mainly expressed in the leaves, whereas *AhMDH12*, *AhMDH11*, *AhMDH2*, *AhMDH13*, and *AhMDH10* were mainly expressed in the roots. Among them, *AhMDH1*, *AhMDH8*, and *AhMDH14* were highly expressed in leaves treated with 300 μM Mn, *AhMDH13* was highly expressed in roots treated with 300 μM Mn, *AhMDH14* was not expressed in normal leaves, and *AhMDH15* was not expressed in leaves treated with 300 μM Mn. Overall, 15 *AhMDHs* were expressed differentially in both peanut leaves and roots.

### 3.8. AhMDH Gene Expression Analysis under Mn Stress

**Figure 7:**
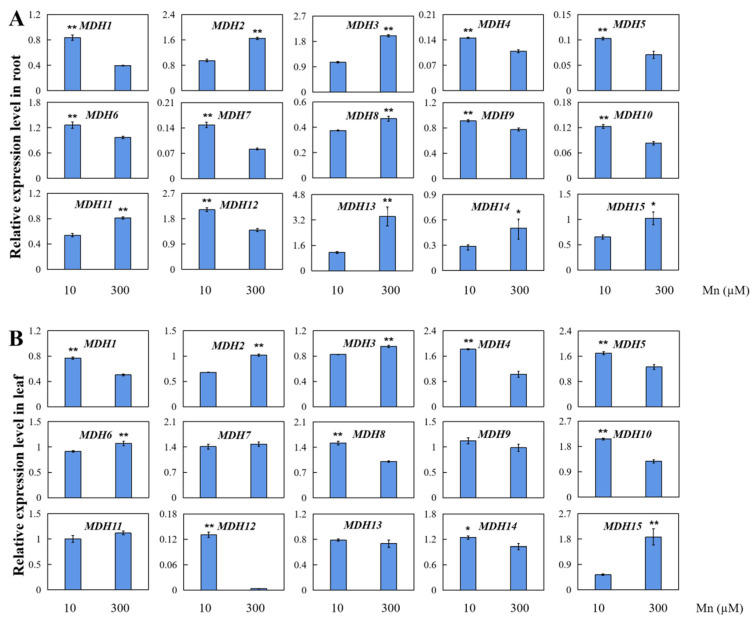
qRT-PCR detection of 15 *AhMDHs* in peanut leaves and roots at concentrations of 10 and 300 µM manganese. Relative expression levels of *AhMDHs* in peanut (**A**) roots and (**B**) leaves. The data are represented by the mean and standard deviation of the four experimental replicates. *T*-test was used to assess the difference between control group and manganese poisoning (* *p* < 0.05, ** *p* < 0.01).

*AhMDHs* in peanut leaves and roots after Mn treatment were analyzed using qRT-PCR, and the results of the gene expression of those 15 *AhMDHs* in roots and leaves after Mn treatment were further verified (Figure 7). All 15 genes showed different degrees of tissue-specific expression in all tissues. When peanuts sustained Mn toxicity stress, they displayed various expression patterns in roots and leaves. All 15 *AhMDHs* in the peanut roots were significantly upregulated or downregulated. Nevertheless, the expression of *AhMDH7*, *AhMDH9*, *AhMDH11*, and *AhMDH13* in leaves under Mn toxicity stress had no significant difference.

## 4. Discussion

With the development of bioinformatics technology, an increasing number of *MDH* members have been authenticated in plants, such as *Oryza sativa* [15], *Glycine max* [45], and *Stylosanthes guianensis* [18]. Eight MDH genes in beans (*Phaseolus vulgaris*) have been identified and found to be mainly located in the cytoplasm and chloroplasts [1]. Seven SgMDHs were found in stylosanthes, and subcellular localization showed that SgMDH mainly exists in the cytoplasm, chloroplasts, peroxidase bodies, and vacuolar membranes [18]. In addition, 12 *OsMDHs* have been confirmed in *O. sativa* [15], and 12 *SlMDHs* have also been verified in tomato (*Solanum lycopersicum*) [17]. Twenty *MpMDHs* have been identified in the whole genome of apple (*Malus pumila*) [18], and studies have shown that the number of *MDHs* is different in different species of plants and that the *MDHs* are distributed in different subregions of plant cells. In this study, 15 *AhMDHs* were identified in peanut, and they were all located in the mitochondria and chloroplasts. Chloroplasts and mitochondria are important organelles for energy metabolism [46]. This indicates that peanut *AhMDHs* might participate in regulating energy metabolism, regulating peanut growth, and responding to abiotic stress.

The analysis results of conservative motifs showed that there were basically 8–9 conserved motifs in all MDH proteins (Figure 4), indicating that the AhMDH protein has a highly conservative protein composition. Those findings agree with the result of MDH in poplar (*Populus trichocarpa*) [19] and cotton (*Gossypium hirsutum*) [47], suggesting that the MDHs are relatively conservative in the long process of natural selection and species evolution. The comparison with MDHs in various plants showed that MDHs experience wide extension during evolution [14,15].

AhMDH cis-acting factor interpretation revealed different plant development factors and stress response factors (Figure 5). Many cis factors were found in the *AhMDHs*, such as MYB binding site respond factors, auxin respond factors, SA (salicylic acid) respond factors, low-temperature respond factors, endosperm expression factors, meristem expression elements, erythromycin response elements, zein metabolism regulation elements, stress and defense respond factors, circadian rhythm control factors, and cell cycle regulation factors. This suggests that *AhMDHs* play a vital regulatory part in response to hormone synthesis, plant growth, biotic and abiotic stresses, and so on.

Gene expression profiles provide a crucial basis for confirming the function of genes [48]. A few *MDHs* have been proven to be specifically expressed in some plant tissues and to play vital roles in the development of seeds [49], root growth [50], light respiration [51], and stress resistance [46]. In the present study, various expression profiles of *AhMDHs* were discovered in the roots and leaves of peanut. Furthermore, all of the *AhMDHs* in peanut leaves and roots were found to be differentially expressed. These findings imply that *AhMDHs* may play a vital regulatory role in peanut root and leaf development.

Plant MDHs are essential for seed development, photosynthesis, abiotic stress tolerance, and biological stress response [14]. MDH also plays a crucial part in plants under heavy-metal stress [15,52]. When plants are subjected to heavy-metal stress, MDH can catalyze the change from oxaloacetic acid to malate and increase the content of malate [15,52]. Some studies indicate that organic acids such as malate can interact with heavy metals to transform them into non-poisonous or low-toxicity combined states, decreasing the poisonous results of heavy metals in plants [18,52]. It has been found that MDH is involved in the differential regulation of various metal ions [53]. Studies on the leguminous crop Stylosanther showed that, under the stress of different heavy metals (iron, manganese, cuprum, zinc, aluminum, cadmium, and lanthanum), *SgMDH* expression levels were different, indicating that SgMDH has different response functions under different metal stresses [18]. A study on SgMDH1 in *S. guianensis* showed that the plant can alleviate manganese stress in soil by enhancing the content of endogenous malic acid and promoting the secretion of malic acid, thus enhancing the Mn tolerance of the plant [54]. Some research reports suggest that rice OsMDH may enhance plant stress resistance through the metabolic pathway of salicylic acid [14], and the overexpression of *OsMDH* can improve the stress tolerance of rice to iron [55]. *mMDH2* participates in cadmium toxic stress in *A. thaliana*, and the *mMDH2* regulates cadmium resistance negatively by regulating ROS (reactive oxygen species)-mediated signaling and ROS content [56]. Further studies have found that Arabidopsis plants overexpressing *mMDH2* show a tolerant phenotype under iron-deficient conditions, and it is suggested that *mMDH2* may mediate the expression of *NAS4* to indirectly respond to iron deficiency stress [57]. In plants, manganese is essential for respiration, protein synthesis, hormone biosynthesis, and photosynthesis [32]. Mn toxicity occurs when the available Mn content in the soil exceeds the typical Mn concentration required by the plant. Plants show evident evidence of poisoning on their leaves as a consequence, such as dark spots, crinkled leaves, and chlorosis; Mn poisoning also affects roots, resulting in a decrease in lateral root count and dry weight, in addition to the signs found on the leaves [33]. The findings in this study revealed that *AhMDHs* are differentially expressed in the roots and leaves of peanut subjected to Mn toxicity stress. These results indicated that *AhMDHs* might play a significant regulatory role in peanut when responding to Mn toxicity stress. The specific molecular regulatory mechanisms involved are unclear and require further research.

## 5. Conclusions

In the present study, 15 *AhMDHs* in the genome of peanut were identified using multi-analysis. The physical and chemical properties, gene structure, phylogeny, and gene expression modes of *AhMDHs* were verified. The phyletic evolution of the 15 *AhMDHs* were separated into three categories. Based on the phylogeny tree interpretation, *AhMDHs* were only included in groups I and III, implying that those peanut MDH genes might play different roles in comparison with MDH in other species. The conservative motifs, protein interaction regulatory networks, and gene structure of *AhMDHs* were of high conservation, indicating that they were functionally conserved.

The cis-element analysis of the AhMDH gene revealed that all 15 *AhMDHs* were participated in regulation of stress response, hormone synthesis, and photosynthesis. The gene expression profiles of the *AhMDHs* based on transcriptome sequencing results revealed various gene expression patterns in the peanut roots and leaves. qRT-PCR testing results demonstrated that *AhMDH* expressions in peanut roots and leaves were upregulated or downregulated under high Mn stress. These consequences lay the foundations for the thorough elucidation of *AhMDH* functions in peanut signal transduction in response to high Mn stress.

## Figures and Tables

**Table 1:** Summary of basic information of *AhMDHs*.

Primer Name	Gene ID	Chr (Arahy)	CDS (bp)	Protein Length (aa)	MW (kDa)	Isoelectric Point (PI)	Subcellular Predicted
*AhMDH1*	LOC112790043	03	1038	345	35.98	9.00	Mitochondrion
*AhMDH2*	LOC112718423	10	1359	452	47.55	8.37	Chloroplast; mitochondrion
*AhMDH3*	LOC112776877	19	1314	437	47.85	6.10	Chloroplast
*AhMDH4*	LOC112791339	03	1071	356	37.44	7.64	Chloroplast; mitochondrion
*AhMDH5*	LOC112711210	09	999	332	35.67	6.11	Chloroplast; mitochondrion
*AhMDH6*	LOC112728667	12	1071	356	37.50	6.97	Chloroplast; mitochondrion
*AhMDH7*	LOC112776383	19	999	332	35.63	6.11	Chloroplast; mitochondrion
*AhMDH8*	LOC112800443	05	1083	360	37.90	7.62	Chloroplast; mitochondrion
*AhMDH9*	LOC112711491	09	1314	437	47.91	6.10	Chloroplast
*AhMDH10*	LOC112737907	13	1038	345	36.10	8.89	Mitochondrion
*AhMDH11*	LOC112735613	02	984	327	35.45	6.09	Chloroplast; mitochondrion
*AhMDH12*	LOC112726743	12	984	327	35.43	6.01	Chloroplast; mitochondrion
*AhMDH13*	LOC112710233	01	1239	412	43.40	8.17	Chloroplast; mitochondrion
*AhMDH14*	LOC112711212	09	999	332	35.66	6.11	Chloroplast
*AhMDH15*	LOC112722502	11	1239	412	43.32	8.47	Chloroplast; mitochondrion

## Data Availability

Data are contained within the article and Supplementary Materials.

## Supplementary Materials

The following supporting information can be downloaded at: https://www.mdpi.com/article/10.3390/genes14122109/s1, Table S1: FPKM values of AhMDHs in peanut roots and leaves under manganese toxicity stress treatment derived from RNA sequencing; Table S2: Primers of AhMDHs family for qRT-PCR detection.
