# Supplementary material for: Genome-Wide Identification of AhMDHs and Analysis of Gene Expression under Manganese Toxicity Stress in Arachis hypogaea
Source: Genes (Basel). 2023 Nov 21;14(12):2109. doi: 10.3390/genes14122109 (PMC10743186; doi:10.3390/genes14122109)
Supplement: Supplementary file 1 [file genes-14-02109-s001.zip › Table S1.pdf]

Table S1. FPKM values of *AhMDHs* in peanut roots and leaves under manganese toxicity stress treatment derived from RNA sequencing

	Log10 (FPKM)			
Gene ID	Leaves-300	Roots-300	Leaves-CK	Roots-CK
AhMDH1	1.84	1.53	1.74	1.54
AhMDH2	0.28	1.22	-0.53	0.92
AhMDH3	1.88	0.87	1.97	0.98
AhMDH4	2.12	1.03	2.24	1.12
AhMDH5	1.84	1.28	1.93	1.17
AhMDH6	2.15	1.44	2.28	1.47
AhMDH7	1.77	1.24	1.83	1.21
AhMDH8	1.13	1.07	0.99	1.05
AhMDH9	1.96	1.14	2.04	1.09
AhMDH10	1.43	1.37	1.27	1.39
AhMDH11	0	0.35	0	0.34
AhMDH12	0	0.37	0	0.42
AhMDH13	1.02	1.46	0.83	1.22
AhMDH14	1.66	1.36	1.47	1.39
AhMDH15	0.66	0.69	0.7	0.6

Notes: 300: 300 μM Mn; CK: 10 μM Mn.
