# Supplementary material for: Genome-Wide Identification of AhMDHs and Analysis of Gene Expression under Manganese Toxicity Stress in Arachis hypogaea
Source: Genes (Basel). 2023 Nov 21;14(12):2109. doi: 10.3390/genes14122109 (PMC10743186; doi:10.3390/genes14122109)
Supplement: Supplementary file 1 [file genes-14-02109-s001.zip › Table S2.pdf]

Table S2 Primers of *AhMDHs* family for qRT-PCR detection

Primer name	Gene ID	Forward Sequence (5' to 3')	Reverse Sequence (5' to 3')
AhMDH1	LOC112790043	TAGCCCTGTCTCCACTGCAT	TACACACAGGCCGCAATAGG
AhMDH2	LOC112718423	CCGTTGGAATCACCCACAGA	TTGGGACTCCAATGTCACGG
AhMDH3	LOC112776877	CATCCAAGGCACAAACGCAA	GCAATTATCAGCCACAGCCG
AhMDH4	LOC112791339	AAGATCGTCGGCTGCATCAA	TTCGATCGGCATGGCATACT
AhMDH5	LOC112711210	CACCGGGATTCAAGGTAGCA	TTTCCTTGGGACACCAGCAG
AhMDH6	LOC112728667	TGAGTCGTCTCTTCGTGTGC	CACTCGTACACGTCCCCATC
AhMDH7	LOC112776383	CCGGAGTGGTGGAATGTTCT	CGCGACCAAGACGTACCTT
AhMDH8	LOC112800443	CAGGGCAAATCGGGTATGCT	TGCATGCCTCAACCACATCA
AhMDH9	LOC112711491	ATTTTTGGGGGCAAGCCAAC	AGCAATTGGAACCGTGGAGT
AhMDH10	LOC112737907	ATGGAGCGTGCTGACTTGTT	TGCCCCAGATTGTCACGTTT
AhMDH11	LOC112735613	TTGCTGGGGTCAATGTACCG	TCCGGCATAAGCCATTGACA
AhMDH12	LOC112726743	TCAAGGAGTTTGCTCCGTCC	GTTGAGCCATTCATCGTCGC
AhMDH13	LOC112710233	ACCGTTGGAACTGCCCATAG	TTGCGTTTGTGCCTTGGATG
AhMDH14	LOC112711212	TCTTGAAAAGCATGCCGCTG	TCATCAACCACAAGCTCCCG
AhMDH15	LOC112722502	TCGCAGGTCAGGGATTTCAC	TGAAACTCGCAGAGGGCTTT
